# Use of Radioisotope Ratios of Lead for the Identification of Historical Sources of Soil Lead Contamination in Santa Ana, California

**DOI:** 10.3390/toxics10060304

**Published:** 2022-06-03

**Authors:** Shahir Masri, Alana M. W. LeBrón, Michael D. Logue, Patricia Flores, Abel Ruiz, Abigail Reyes, Juan Manuel Rubio, Jun Wu

**Affiliations:** 1 Department of Environmental and Occupational Health, Program in Public Health, University of California, Irvine, CA 92697, USA; mdlogue@uci.edu; 2 Department of Health, Society, and Behavior, Program in Public Health, University of California, Irvine, CA 92697, USA; alebron@uci.edu; 3 Department of Chicano/Latino Studies, University of California, Irvine, CA 92697, USA; 4 Orange County Environmental Justice, Santa Ana, CA 92705, USA; patricia@ocej.org; 5 Jóvenes Cultivando Cambios, Santa Ana, CA 92705, USA; agruiz@ucdavis.edu; 6 Community Resilience, University of California, Irvine, CA 92697, USA; abigail.reyes@uci.edu; 7 School of Humanities, University of California, Irvine, CA 92697, USA; rubiojm1@uci.edu

**Keywords:** urban soil, Pb exposure, environmental justice, lead isotope, heavy metal

## Abstract

Lead (Pb) is an environmental neurotoxicant that has been associated with a wide range of adverse health conditions, and which originates from both anthropogenic and natural sources. In California, the city of Santa Ana represents an urban environment where elevated soil lead levels have been recently reported across many disadvantaged communities. In this study, we pursued a community-engaged research approach through which trained “citizen scientists” from the surrounding Santa Ana community volunteered to collect soil samples for heavy metal testing, a subset of which (n = 129) were subjected to Pb isotopic analysis in order to help determine whether contamination could be traced to specific and/or anthropogenic sources. Results showed the average ^206^Pb/^204^Pb ratio in shallow soil samples to be lower on average than deep samples, consistent with shallow samples being more likely to have experienced historical anthropogenic contamination. An analysis of soil Pb enrichment factors (EFs) demonstrated a strong positive correlation with lead concentrations, reinforcing the likelihood of elevated lead levels being due to anthropogenic activity, while EF values plotted against ^206^Pb/^204^Pb pointed to traffic-related emissions as a likely source. ^206^Pb/^204^Pb ratios for samples collected near historical urban areas were lower than the averages for samples collected elsewhere, and plots of ^206^Pb/^204^Pb against ^206^Pb/^207^ showed historical areas to exhibit very similar patterns to those of shallow samples, again suggesting lead contamination to be anthropogenic in origin, and likely from vehicle emissions. This study lends added weight to the need for health officials and elected representatives to respond to community concerns and the need for soil remediation to equitably protect the public.

## 1. Introduction

Lead (Pb) is an environmental neurotoxicant that has been associated with a wide range of adverse socioeconomic conditions and educational and health outcomes [1,2]. Historically, low-income communities and communities of color, as well as residents of urban areas, have been disproportionately impacted by Pb exposure [3,4,5]. What is more, children, given their increased lead exposure (e.g., greater hand-to-mouth activity, lead in play areas, etc.), can experience attention-deficit/hyperactive disorder, behavioral issues, declines in IQ, lowered test scores, and other developmental issues when they experience blood-lead levels of 10 μg/dL or lower [6,7,8,9]. Further, even low blood-lead levels are associated with adverse health outcomes over the life course.

In community environments, lead contamination of the soil is persistent due in part to limited degradation of lead, minimal disturbances of the soil, and limited systemic efforts to remediate soil lead. While a robust body of literature suggests that no level of Pb exposure is safe for young children [1], the U.S. Environmental Protection Agency (EPA) has established a limit of soil Pb concentrations in the soil of 400 parts per million (ppm) in areas where children play, and 1200 ppm in other uncovered areas [10]. However, to better protect children, the California EPA’s Office of Environmental Health Hazard Assessment advises that areas where children play should have soil Pb concentrations at or below 80 ppm [11].

To better understand residential environmental contamination by lead and other pollutants where government agency data is absent, collaborations have increasingly been forming between scientific researchers and community leaders and residents in order to design and implement community-engaged participatory research methods that foster a healthier environment. These methods involve community members, including racially marginalized groups (e.g., indigenous, African American, and Latina/o/x communities), in each step of the research process, including the development of research aims, data collection and the dissemination of results so as to facilitate increased accessibility, inclusivity and democratization of scientific information [12,13,14,15,16].

In our prior work that engaged volunteer “citizen scientists” from the community to facilitate the collection of soil samples, we found soil lead concentrations in the city of Santa Ana, California, to exceed the 80-ppm recommendation in 751 of 1528 (48%) soil samples, and to exceed the 400-ppm standard in 60 (4%) of samples [15]. Elevated lead levels were disproportionately present in census tracts with higher proportions of lower-income and Latina/o/x communities. Since the publication of our findings, Santa Ana residents have not only begun to advocate for remediation strategies and lead-prevention measures that will be equitable, effective, and community-based, but also to raise central questions surrounding the origin of the city’s lead contamination and whether it can be traced back to anthropogenic emission sources (e.g., historical vehicle emissions, lead paint, etc.), or whether the lead is simply an artifact of naturally high crustal levels.

In general, sources of soil Pb contamination include both historical and present-day emissions. Prior to its incremental phaseout beginning in the 1980s, and its subsequent ban from on-road use by the EPA in 1996, leaded gasoline and therefore vehicle traffic represented a major source of lead emissions in the United States [17]. Additionally, lead paint was historically used on many houses and other buildings throughout the United States prior to its ban in 1978. Disturbances of these painted surfaces through building renovations, demolitions, and weathering over time is therefore another key contributor to urban soil contamination [18]. In Santa Ana, given that an estimated 81% of housing units were constructed in the 1970s and earlier, and that the city is bordered by three major freeways and an airport, both vehicle emissions and lead paint represent likely sources of soil lead contamination [19]. Lead emissions from industry (e.g., metal fabrication, metal cutting, metal processing, etc.) and agriculture (e.g., lead arsenate pesticides) represent other potential sources.

A key quantitative technique for the tracing of historical lead pollution to specific sources is to examine the ratios of lead isotopes. The lead isotopes ^206^Pb, ^207^Pb, and ^208^Pb are produced by the radioactive decay of ^238^U, ^235^U, and ^232^Th, which have half-lives of 4.5, 0.7, and 14 billion years, respectively. ^204^Pb has no known radioactive progenitor and is therefore considered a stable measure of the original lead that existed when the elements were first formed, sometimes called “primeval lead.” Since the formation of Earth, the three radiogenic lead isotopes have followed a smooth increase in abundance at known independent rates relative to the non-radiogenic isotope ^204^Pb. Therefore, the observed isotopic ratios of various soils and other media will depend, at least in part, on the Th/Pb and U/Pb ratios of the source rock and the age of the ore, allowing such compositions to serve as isotopic “signatures” that allow for the tracing of environmental lead pollution back to its source. This approach to understanding lead source attribution has been applied in prior studies to distinguish between lead originating from leaded gasoline emissions, coal combustion, etc. [20,21,22,23].

In this study, we build upon such work, as well as the robust history of community-academic partnerships in environmental health research, and our previous work in Santa Ana, by combining an analysis of lead isotopes and enrichment factor (EF) values from a subset of 129 soil samples to help answer pressing questions by residents as it relates to the origins of the lead soil contamination in their city [15,24]. Specific research questions included: (1) Can lead isotopic ratios be successfully applied in combination with EF values to identify whether lead soil contamination in Santa Ana is anthropogenic in origin? (2) Can lead isotopic ratios, combined with EFs, help to distinguish between lead originating from historically combusted leaded gasoline and historical lead paint? (3) Can historical urban features aid in our ability to attribute lead contamination to anthropogenic sources? Our hypothesis was that such techniques could indeed be applied to identify the anthropogenic nature of soil contamination in the city, as well as determine the dominant anthropogenic sources from which soil Pb originated.

The novelty of this work was the community-driven nature of the research questions and innovative approach to community-based participatory data collection which engaged local residents to facilitate the collection of soil samples. What is more, we applied a novel approach to examining lead isotopic data using a recently developed, innovative method derived from archival material of historical urban features, such as historical road maps and historical aerial images, and compared isotopic ratios near such features with those of contemporary features. The importance of this work is underscored by the fact that many cities around the country are similarly composed of both historical and contemporary areas, each having undergone similar histories as it relates to development and potential contamination by lead. Thus, the methods applied in this study and our ultimate findings are likely applicable elsewhere.

## 2. Materials and Methods

This study is the result of a multi-year community-academic collaboration that was established following the publication of a report showcasing lead pollution within the city of Santa Ana, California [25]. Orange County Environmental Justice (OCEJ) teamed up with the youth collective Jóvenes Cultivando Cambios (JCC; Youth Cultivating Changes), along with scientists and staff at the University of California, Irvine (UCI), to initiate a campaign (¡Plo-NO Santa Ana! or Lead-Free Santa Ana!) focused on investigating the lead contamination issue in this city and advocating for its remediation, which culminated in a comprehensive soil pollution study involving the collection of soil samples across Santa Ana, CA [2]. The analysis of social and spatial distributions of soil-lead in Santa Ana, and the health risks they present, were subsequently published [15,26]. However, an important question that went unanswered by these previous investigations, yet which was frequently asked by Santa Ana residents and OCEJ members, was “Where did all this lead come from?” The present study stems from this unanswered question and the important concern among community members about the sources of lead contamination in their city.

This research is connected with collaborative efforts to remediate the lead crisis in Santa Ana and address environmental injustices in both Orange County and elsewhere. It contributes to strengthening community-science models of organization, which strive to leverage peer-reviewed studies for mobilizing residents, conducting community science (in which members of impacted communities lead the research agenda and become actively involved in the research process), and supporting public advocacy efforts related to education, outreach, policy, and remediation [27,28,29]. This research directly informs policy debates and recommendations, as well as resident-driven discussions regarding the establishment of a healthier environment. Data for the analyses described below are drawn from archival sources and soil samples collected by our trained personnel and from the U.S. Census Bureau’s American Community Survey. The UCI Institutional Review Board classified this study as exempt.

### 2.1. Study Region

Santa Ana is a densely populated city located in southern California in the southwestern region of the United States. It is the administrative center of Orange County, which is the sixth most populated county in the U.S. With a total population of approximately 337,716 residents, Santa Ana spans an area of 70.6 km^2^ and includes 61 Census tracts [30]. In terms of population, Santa Ana ranks the second largest city in Orange County, and is the eleventh largest city in the state [30]. The majority of Santa Ana residents identify as Latina/o/x (77.3%), followed by Asian (11.4%) and white (9.4%), with a relatively high proportion (45.2%) of residents being immigrants [31]. As of 2019, the city includes 78,563 housing units and has a median household income of USD 65,313 (2018 dollars) [30].

### 2.2. Field Sampling

In the summer and fall of 2018, trained community members collected soil samples across various land-use types that included parks, residential areas, industrial zones, etc. Field teams responsible for soil sampling and the recording of land-use types consisted of local community members, including numerous members of the youth community, who were first trained by a field coordinator.

Building upon methods by Wu et al. (2010), field teams selected sampling locations at each sampling site that were not obstructed by physical barriers [32]. Where possible, field teams marked a three-foot radius and obtained soil samples from five distinct points (one central point and four other points that were three feet away from the central point). Soil samples were collected at a depth of 2 to 6 cm after first removing 1 cm of soil (including vegetative matter). Both shallow (2–6 cm) and deep (10–20 cm) soil samples were collected. Samples were air dried and sieved with brass screen (#50 mesh, twice; #100 mesh once), yielding fine soil dust samples to characterize lead exposures for which young children are most vulnerable [33]. In total, 1528 samples were obtained throughout Santa Ana, resulting in a highly spatially resolved characterization of soil heavy metals. To establish a baseline soil Pb level, eight soil samples were collected outside of Santa Ana in nearby state and regional parks in Orange County that could be considered relatively pristine and unaffected by major local anthropogenic lead sources (i.e., traffic, industry, buildings). A subset of 129 soil samples that included samples from a diverse range of land-use types, covering both shallow (n = 110) and deep (n = 19) samples, with representative geographic distributions, and containing both very low and very high lead concentrations were selected for lead isotopic analysis. Baseline samples (n = 8) were also included for isotopic analysis. This subset of soil samples (n = 129), which includes the eight baseline samples, is the focus of the present analysis.

### 2.3. Soil Analysis

For the lead isotope analysis, all sample preparation work (except weighing) was done in a Class 1000 or Class 100 cleanroom at the University of Georgia Institute for New Materials Research. All reagents used were Optima or Suprapure grade, and all water was 18 MΩ Millipore. Soil samples were baked in pre-cleaned alumina crucibles for 5 h at 375 °C to drive off organic carbon, a step which was conducted to prevent the impairment of cation exchange and acid dissolution (for quality control, a small subset of samples analyzed with and without this step demonstrated nearly identical Pb isotope results). Then, in a class 100 laminar flow bench, each sample was transferred to an agate mortar and pestle (cleaned between each sample) and disaggregated, transferred back to its original 45 mL centrifuge tube, and homogenized by shaking. Subsamples, each weighing approximately 50 mg, were then taken from each soil sample, and Pb was extracted.

Lead extraction was carried out by a method that consisted of full dissolution for 48 h in a sealed, clean Savillex dissolution beaker containing a mixture of concentrated HF:HNO_3_. This method follows that of Weis et al. (2006) [34]. Samples were allowed to sit overnight at room temperature, and subsequently centrifuged for 15 min at 13,000 rpm. The acid was then carefully decanted or pipetted into a clean Savillex beaker. After these steps, samples were dried on a hotplate, taken back into solution in concentrated HNO_3_, dried a second time, and finally taken into solution in 500 microliters of HBr. A 100-microliter aliquot of this was then pipetted onto a cleaned PTFE column containing a bed of Dowex 1 × 8 resin and Pb was separated by standard chromatographic techniques for Pb separation (e.g., following the protocol of Simbo et al., 2019) [35].

Measurement of Pb isotope ratios was carried using a Nu II MC-ICP-MS (Nu Instruments, Wrexham, U.K.) fitted with a Cetac Aridus II desolvenator (dry mode). This configuration decreases interferences and increases sensitivity. Sample concentrations were kept sufficiently high so as to obtain at least 100 millivolt of signal on the least abundant Pb isotope (^204^Pb) in order to minimize measurement error. Process blanks were monitored by measuring signal intensity on all cups and were in the 10^−3^ to 10^−4^ range. Immediately prior to analysis, all samples and standards were spiked with a Tl solution (Pb:Tl~5:1) for mass-fractionation correction. Throughout the data collection process, NBS 981 Pb standard was run before and after each sample to monitor instrument stability and to accommodate normalization to published values of NIST 981 (see below). All results were corrected for isobaric interference from ^204^Hg, then empirically normalized using the exponential law for mass bias correction using ^205^Tl/^203^Tl = 2.3875, as described by Belshaw et al. (1998) [36]. Over the course of measurement, standard errors for the isotope ratios of ^208^Pb/^204^Pb, ^207^Pb/^204^Pb, ^206^Pb/^204^Pb, ^208^Pb/^206^Pb, and ^207^Pb/^206^Pb were ±0.018 (0.08%), ±0.005 (0.06%), ±0.014 (0.14%), ±0.0011 (0.05%), and ±0.0006 (0.07%), respectively.

To measure general soil lead concentrations (as opposed to isotopes), samples were analyzed via X-Ray Fluorescence (XRF) instrumentation (SPECTRO XEPOS HE Benchtop XRF Spectrometer), a well-established and recognized method for identifying the total lead levels [37]. Each soil sample was scanned five times by the XRF machine to ensure reproducibility and stability of measurements, showing a low average absolute measurement error of 1.0% across all Pb samples. To further confirm quality laboratory analysis, a subgroup of samples (n = 18) was subjected to XRF analysis a second time (five more scans), yielding an excellent correlation (r = 1.0). Further details regarding XRF analysis can be found in our prior work [15,26].

### 2.4. Historical and Landuse Variables

This study leveraged historical analyses that drew upon the work of historians of the United States and Orange County to better understand potential differences in isotopic composition between lead in historical and contemporary areas, and therefore to enable inferences about lead source contributions to the environment. It also drew from original archival research recently conducted at the Orange County Archives in Santa Ana, as well as online collections, such as the David Rumsey Map Collection, the Online Archive of California, and the historical aerial image collection at Orange County Public Works. Archival material was used to generate data for scientific analysis. This methodological approach largely involved the examination of historical maps and aerial photographs produced between 1906 and 1980, which were digitized, georeferenced, and ultimately imported into ArcGIS software as shapefiles (or layers). Examples of such layers included polylines and polygons drawn to reflect historical urban features such as surface streets and the extent of urban development that existed over discrete periods in history (e.g., 1906, 1938, 1960). Shapefiles were then overlaid with the contemporary city map containing the locations of the points where soil-lead isotopic measurements were collected.

In terms of contemporary data, this analysis considered five current land-use types including: arterial roadways, schools, parks, industrial areas, and residential areas. Garden areas were consolidated into the “parks” category for simplicity and to maintain reasonable sample sizes for categorical analysis. Further details on how land-use types were identified and categorized can be found in our prior work [15].

### 2.5. Enrichment Factor

An enrichment factor (EF) is a geochemical tool that is commonly used to examine the extent of heavy metal pollution of the soil [38]. It is defined as follows:

(1) $$\mathrm{EF}={\left(\frac{C_{n}}{C_{\mathrm{ref}}}\right)}_{\mathrm{sample}}÷\;\;\;\;{\left(\frac{B_{n}}{B_{\mathrm{ref}}}\right)}_{\mathrm{background}}$$

where (C_n_/C_ref_) _sample_ is the concentration ratio of a particular metal and a particular reference element in a sample, and (B_n_/B_ref_) _background_ represents the ratio of the natural baseline concentration of that metal relative to the reference element. Elements most commonly utilized as reference elements include conservative elements such as iron (Fe) and aluminum (Al) [39]. In this study, both Fe an Al were used and compared as reference elements. Baseline values used to calculate EF values were derived from the eight baseline samples collected outside of Santa Ana that were described previously.

To decipher EF values, we applied a five-category system introduced previously by Sutherland and employed in other studies [39,40], which indicates: no or minimal enrichment (EF < 2), moderate enrichment (2 ≤ EF < 5), significant enrichment (5 ≤ EF < 20), very high enrichment (20 ≤ EF < 40), and extremely high enrichment (EF ≥ 40).

## 3. Results

**Figure 1:**
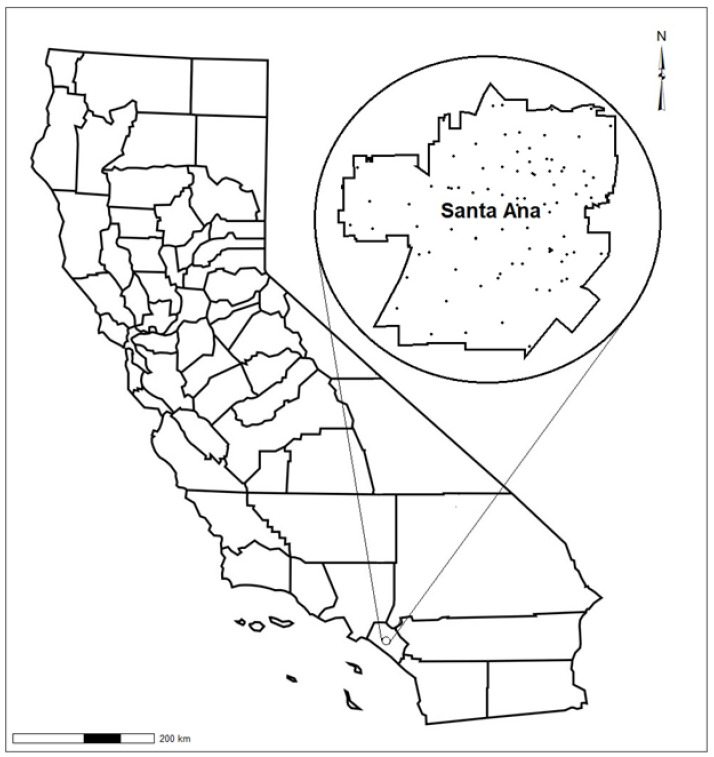
Study location and isotope samples sites.

Figure 1 depicts the locations where the lead isotope samples (n = 129) were collected in Santa Ana, CA. As shown, the soil samples chosen for isotopic analysis included those from a wide range of areas of the city, resulting in a spatially homogenous survey of the city.

**Figure 2:**
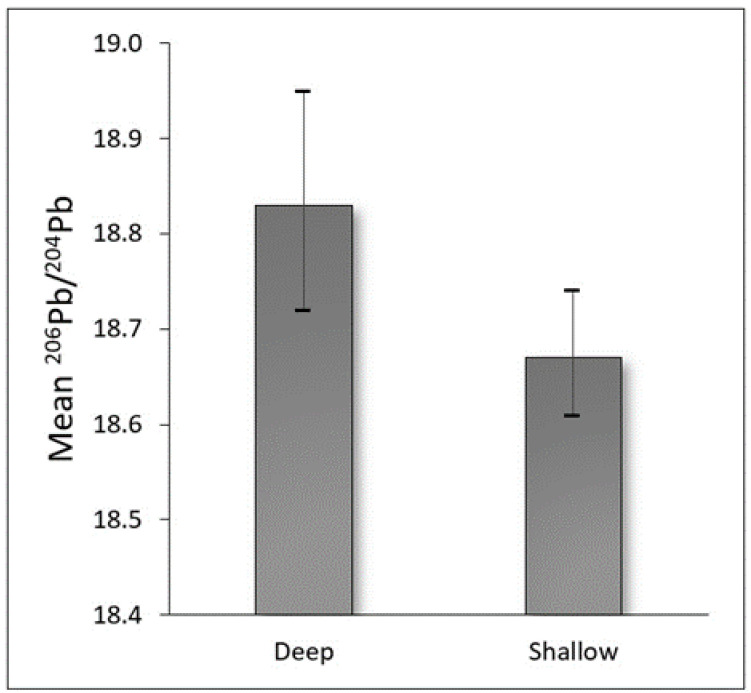
Average ^206^Pb/^204^Pb ratios (with 95% confidence intervals) of shallow and deep soil lead samples.

Figure 2 presents a comparison of shallow and deep soil lead samples as it relates to the ^206^Pb/^204^Pb ratio. A statistically significant (*p* < 0.05) difference in the average ^206^Pb/^204^Pb ratio was apparent, with the shallow samples exhibiting a low average value (18.69) consistent with that often reported for historically measured gasoline and particulate emissions, relative to deep samples (18.83).

**Figure 3:**
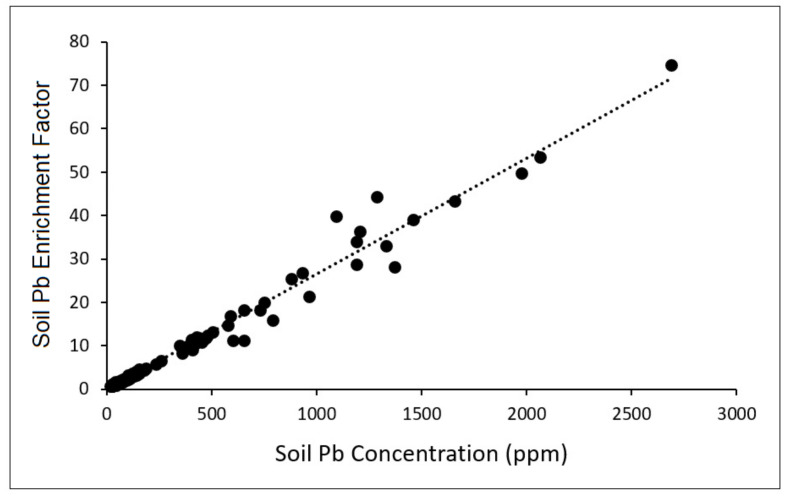
Scatter plot of soil enrichment factors and soil lead concentrations.

**Figure 4:**
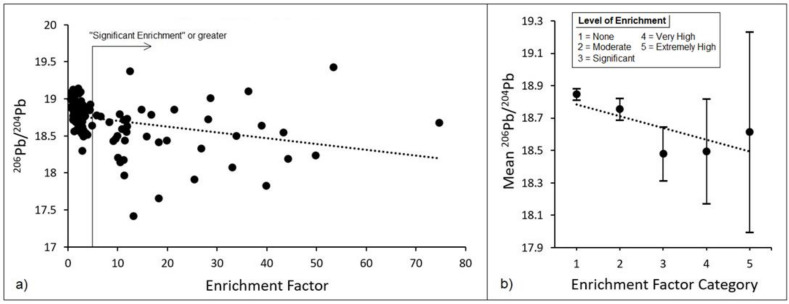
Ratio of (**a**) ^206^Pb/^204^Pb plotted against Pb enrichment factors as measured in the soil, as well as (**b**) average ^206^Pb/^204^Pb (with 95% confidence intervals) plotted against Pb enrichment categories.

Figure 3 presents soil Pb enrichment factors plotted against lead concentrations. As shown, lead enrichment showed a strong positive correlation with absolute lead concentrations as measured in the soil, with the highest lead concentrations showing over 50-times enrichment of lead compared to baseline samples. In Figure 4a, soil Pb enrichment factors exhibited a moderate inverse correlation with soil ^206^Pb/^204^Pb ratios. In particular, the ^206^Pb/^204^Pb ratio was greater than 18.5 for nearly all soil samples where the lead enrichment was considered “significant” or greater. Figure 4b depicts a similar plot, except that enrichment factors have been consolidated into their respective EF value categories, with the associated ^206^Pb/^204^Pb ratio reported as the average (with 95% confidence intervals) across samples within each EF category. A strong negative correlation is observed in this case, although statistical significance was only apparent across the lowest three EF categories and not the higher categories.

**Figure 5:**
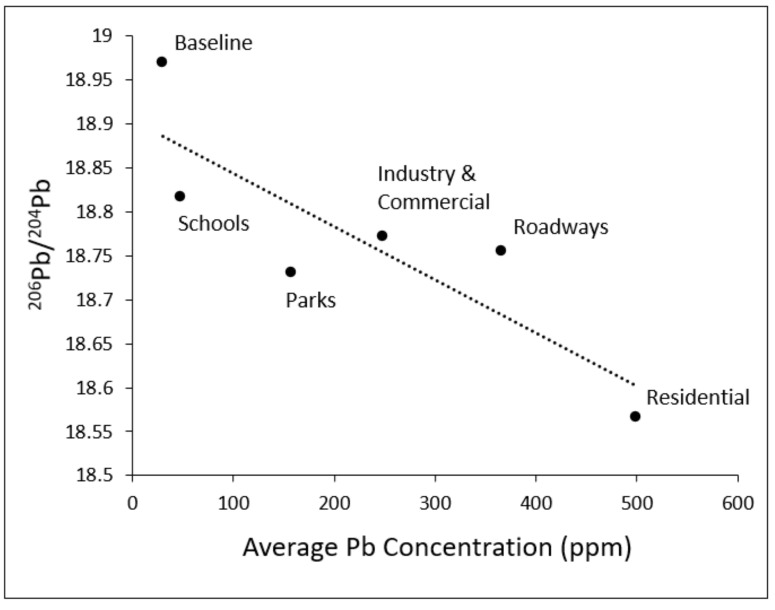
Ratio of ^206^Pb/^204^Pb plotted against absolute Pb concentrations (as measured in shallow soil samples) after averaging by land-use type.

Figure 5 presents the ratio of ^206^Pb/^204^Pb plotted against Pb concentrations (as measured in shallow soil samples) after averaging by land-use type. As shown, an inverse trend consistent with Figure 4 was observed. The residential land-use type exhibited both the highest average lead concentration and lowest average ^206^Pb/^204^Pb ratio, followed by roadways and industry. In contrast, schools and parks, which had the lowest average lead concentrations among Santa Ana land-use types, showed the highest average ^206^Pb/^204^Pb ratios. In general, the ^206^Pb/^204^Pb ratio ranged from 18.57 to 18.83 across Santa Ana land-use types. The average ^206^Pb/^204^Pb ratio for baseline samples (shallow) collected outside of Santa Ana (18.97) was higher than the averages across all five Santa Ana land-use types.

**Figure 6:**
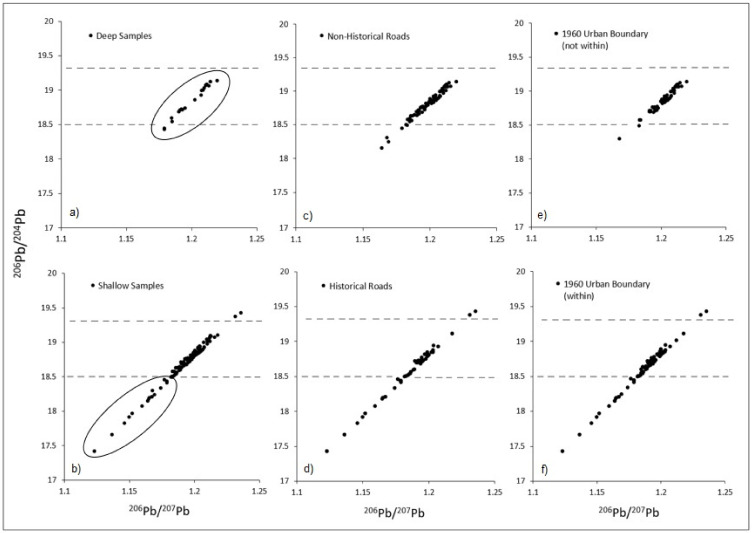
Ratios of ^206^Pb/^204^Pb plotted against ^206^Pb/^207^ for (**a**) deep and (**b**) shallow soil samples, as well as soil samples collected near (**c**) non-historical and (**d**) historical roads, and (**e**) outside and (**f**) within the 1960 urban boundary. Dotted lines represent the approximate lower and upper bounds seen across deep sample clusters (top graphs) to enable visual comparison with shallow samples (lower graphs).

Figure 6 depicts the scatter of ^206^Pb/^204^Pb plotted against ^206^Pb/^207^ for various lead sample groupings. Specifically, Figure 6a,b show a comparison of deep and shallow soil lead samples, respectively, while Figure 6c,d compare non-historical and historical roads and Figure 6e,f compare the scatter for samples collected both outside of and within the 1960 urban boundary, respectively. As shown, deep soil samples exhibited clustering within a relatively narrow range of scatter, whereas the scatter of shallow samples extended much lower toward the lower left quadrant of the plot. The pattern of scatter for deep samples was very similar to that exhibited by non-historical road samples as well as samples collected in the more contemporary areas of the city (not within 1960 boundary). By comparison, samples collected near historical roads and within the more historical parts of the city (within the 1960 urban boundary) exhibited scatters closely resembling that of the shallow samples (where higher anthropogenic source contributions would be expected).

Regarding the linear increases for each radiogenic isotope, this is referred to as the radiogenic growth curve and is consistent with the rate of radioactive decay of its parent. The types of plots shown in Figure 6 are conventional for this type of analysis. By plotting the ratios of the relatively abundant radiogenic isotopes, one can minimize errors that may arise during mass-spectrometry measurements of the less abundant ^204^Pb isotope.

**Figure 7:**
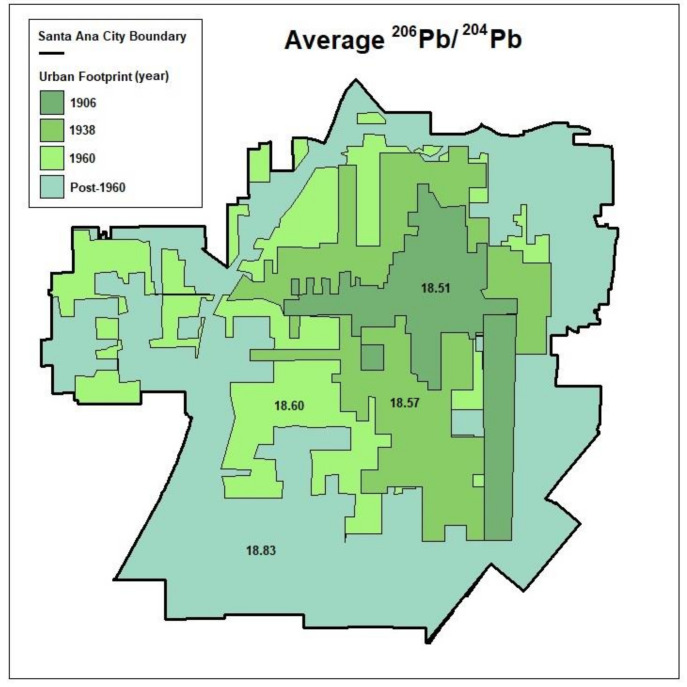
Historical urban footprints in Santa Ana and the average ^206^Pb/^204^Pb ratio across soil samples collected within each region.

Table 1 presents the complete summary of the lead isotope ratio statistics averaged by land-use type and other groupings. While the subgroupings for land-use and depth are mutually exclusive, this is not the case for other subgroupings (e.g., the 1938 urban footprint includes points that are also present in the 1906 footprint). Using data from Table 1, Figure 7 presents the historical urban footprints in Santa Ana and the average ^206^Pb/^204^Pb ratio across soil samples collected within each region. The figure visually illustrates the outward growth of the urban boundary and the corresponding decline in average ^206^Pb/^204^Pb that is observed in the more historical areas. Of note, the full boxplot distribution of the ^206^Pb/^204^Pb ratios across each Table 1 category is presented in Figure S1 of the Supplemental Materials section.

## 4. Discussion

This study examined lead isotopic ratios in a subset of 129 soil samples collected throughout Santa Ana, CA, including nearby baseline samples, in order to determine the sources of urban lead contamination. Ratios were analyzed by calculating averages across various land-use types, soil depths and non-historical features, as well as by examining clustering along the radiogenic growth curve (via scatter plots). Soil lead concentrations and enrichment factors were also explored relative to one another and relative to lead isotopic ratios. The ^206^Pb/^204^Pb ratio was of particular interest in this analysis given prior studies that have reported values for this ratio in aerosols and gasoline (indicators of historical traffic-related emissions).

In general, the ^206^Pb/^204^Pb ratio in shallow soil samples was significantly lower (*p* < 0.05) on average than deep samples. Prior literature suggests low ^206^Pb/^204^Pb ratios (<18.5) to be indicative of historical traffic-related emissions. During the height of leaded gasoline combustion in the 1960s, Chow and Johnstone (1964) reported a ^206^Pb/^204^Pb ratio of 18.04 in aerosols collected in the same general area (Los Angeles Basin) [41]. Similarly, Wayne et al. (1970) found ^206^Pb/^204^Pb to decline with decreasing distance to a major freeway system, reporting an average value of 18.18 obtained from topsoil within 500 feet of the New Jersey Turnpike [20]. Findings regarding the shallow and deep samples collected in the current study are therefore reasonable given that atmospheric deposition would expectedly lead to disproportionate contamination (and a lower ^206^Pb/^204^Pb ratio) in the shallow soil surface relative to the underlying (deep) soil.

When analyzing the average ratio of ^206^Pb/^204^Pb plotted against soil average Pb concentrations by land-use type, an inverse trend was observed in which residential land-use exhibited the highest average lead concentration and lowest average ^206^Pb/^204^Pb ratio, followed by roadways and industry. In contrast, schools and parks had the lowest average lead concentrations and the highest average ^206^Pb/^204^Pb ratios. This inverse trend not only showcases the land-use types with the greatest lead contamination, but also suggests that greater contamination is associated with a greater anthropogenic (i.e., vehicle emissions) contribution. This was consistent with subsequent findings, as discussed below. That residential land-use showed higher lead and a greater anthropogenic footprint may be due to irrigation activities that helped reduce resuspension of soil particles by wind and other physical disturbances over time (gradually carrying them away from a particular site). This hypothesis is supported by our recent work that showed average soil-lead concentrations to be higher in residential environments where irrigation was not present [42].

When examining soil Pb enrichment factors plotted against lead concentrations, a strong positive correlation was observed, with the highest lead concentrations showing over a fifty-fold enrichment of lead relative to baseline samples. This serves as evidence that soil samples with the most elevated lead are not due to natural increases in crustal metals, but rather from anthropogenic sources. Similarly, EF values plotted against ^206^Pb/^204^Pb exhibited a negative correlation. This is a complimentary finding that is consistent with lower ^206^Pb/^204^Pb ratios being associated with historical traffic-related emission sources. Taken together, these findings suggest that lead enrichment in Santa Ana is anthropogenic in origin, and likely originated from vehicle emissions.

Corroborating these findings was our analysis that examined and compared ^206^Pb/^204^Pb ratios across areas categorized as either historical or non-historical. That is, the average ^206^Pb/^204^Pb ratio for samples collected near historical surface streets, historical freeways, and within the historical 1960 urban boundary were on average lower (suggesting anthropogenic/traffic origin) than the averages for samples collected near their more contemporary counterparts. A visual depiction of historical urban footprints clearly illustrates the declining ^206^Pb/^204^Pb ratio with increasing age of the area. Similarly, comparisons of scatter plots between ^206^Pb/^204^Pb and ^206^Pb/^207^ for non-historical areas and more contemporary (post-1960) areas demonstrated a narrow pattern of clustering that was very similar to that of the deep soil samples. In contrast, the clustering of shallow samples exhibited a very similar isotopic composition to that of historical roadways and older areas. Assuming deep samples more closely resemble the natural background lead isotopic signature, and the fact that the general deep soil pattern of isotopic clustering was shared by contemporary urban features but not historical features, collectively reinforces the notion of anthropogenic lead contamination within Santa Ana. Taken together, these findings are reasonable given that the historical areas of the city have experienced a longer history of nearby traffic-related emissions, particularly during the early/mid-20th century era when leaded gasoline was most prevalent.

While our explanation of lower ^206^Pb/^204^Pb ratios near the historical areas of the city is reasonable for the reasons previously explained, it is worth noting that the isotopic composition of environmental Pb pollution in parts of the U.S. has been shown to have decreased toward a less radiogenic composition (^206^Pb/^204^Pb~18.5) during the early phase of industrial growth (pre-1940s) in the U.S., before shifting quickly to more radiogenic compositions (^206^Pb/^204^Pb~18.7) in parallel with the rapid rise in lead emissions following the application of Pb-alkyl additives to gasoline [43]. In general, use of Missouri ores began around 1963 in the United States, which was accompanied by more radiogenic Pb pollution throughout parts of the U.S. [43]. This temporal shift in radiogenic lead pollution in the U.S. may have contributed to the observed difference in the radiogenic compositions reported between historical and less-historical urban areas in this study.

Lead paint represents another potential source of lead contamination in the city. Isotopic compositions of lead paint, however, have been shown to be widely variable. Rabinowitz and Hall (2002), for instance, measured isotopic ratios of six major brands of white lead carbonate paint pigments, and found the isotopic ratios to range dramatically [44]. Even for the National Lead company, which was the dominant producer of white lead between 1936 and 1942 (accounting for ~60% of the market share), the ^206^Pb/^204^Pb ratio ranges from approximately 16.5 to 20 [44]. Similarly, a study measuring ^206^Pb/^204^Pb in lead paint samples collected from homes and classrooms reported ratios that ranged from 17.5 to 18.7 [45]. This makes the attribution of soil lead pollution to historical paint sources challenging. One opportunity to potentially overcome this challenge would be to collect direct paint samples across a wide range of historical Santa Ana homes and other buildings in order to determine an isotopic paint “signature” unique to the Santa Ana area. This, however, was beyond the scope of the present analysis.

This study succeeded in applying a quantitative method to help answer community questions concerning the origin of lead contamination in the city of Santa Ana and the role that historical anthropogenic sources played in accounting for the contemporary spatial distribution of soil lead. Our findings underscore the necessity for public health officials and elected representatives to respond to community concerns and the need for soil remediation to equitably protect public health. Effective methods to reduce community-wide exposure to soil lead, particularly in residential areas afflicted by the highest anthropogenic lead concentrations, include replacing topsoil (or covering topsoil with grass or gravel), bioremediation (e.g., using plants to help remove soil lead), as well as lead paint remediation (where applicable). Given that such measures can be expensive and therefore less feasible for low-income households and/or renters (where landlords control such measures), policy interventions will likely be needed to ensure equitable reductions in community-wide lead exposure and promote environmental justice.

## 5. Conclusions

This study examined lead isotopic ratios in a subset of 129 soil samples collected throughout Santa Ana, CA, including nearby baseline samples, in order to determine the sources of urban lead contamination in the city. Results showed the average ^206^Pb/^204^Pb ratio in shallow soil samples to be lower on average than deep samples, consistent with shallow samples being more likely to experience historical anthropogenic contamination. An analysis of soil Pb enrichment factors plotted against lead concentrations demonstrated a strong positive correlation, further reinforcing the likelihood of lead contamination by anthropogenic activity, while EF values plotted against ^206^Pb/^204^Pb pointed to traffic-related emissions as a likely key source. Corroborating these findings were results showing average ^206^Pb/^204^Pb ratios for samples collected near historical urban areas to be lower than the averages for samples collected elsewhere, as well as similar results demonstrating scatter plots between ^206^Pb/^204^Pb and ^206^Pb/^207^ for historical areas to be very similar to those of shallow samples (in contrast to deep soil samples which showed pattens more similar to soils collected in less historical areas of the city where anthropogenic contamination would be less expected). Taken together, these findings suggest that lead contamination in Santa Ana is largely anthropogenic in origin, and likely originated from vehicle emissions. This study lends added weight to the need for public health officials and elected representatives to respond to community concerns and the need for soil remediation to equitably protect public health.

## Figures and Tables

**Table 1:** Lead concentrations and isotopic ratios averaged by spatial groupings.

		N	Pb (ppm)	^206^Pb/^204^Pb	^207^Pb/^204^Pb	^207^Pb/^206^Pb	^208^Pb/^204^Pb	^208^Pb/^206^Pb	^206^Pb/^207^Pb
**LANDUSE**									
Industrial ^a^		16	248.2	18.77	15.67	0.83	38.55	2.05	1.20
Park		11	157.47	18.73	15.67	0.84	38.48	2.05	1.20
Residential		40	499.6	18.57	15.65	0.84	38.38	2.07	1.19
Roadway		16	387.50	18.76	15.68	0.84	38.53	2.05	1.20
School		3	48.3	18.82	15.71	0.83	38.71	2.06	1.20
**ROADS**									
Non-Freeways		79	312.5	18.71	15.67	0.84	38.52	2.06	1.19
Freeways (All)		31	318.4	18.67	15.66	0.84	38.46	2.06	1.19
Freeways (Historical)		23	365.0	18.64	15.66	0.84	38.42	2.06	1.19
Surface Streets (Non-Historical)		63	203.8	18.79	15.69	0.84	38.62	2.06	1.20
Surface Streets (Historical)		47	462.1	18.58	15.65	0.84	38.35	2.07	1.19
**URBAN FOOTPRINT**									
Post-1960		47	101.1	18.83	15.69	0.83	38.66	2.05	1.20
1960		63	473.1	18.60	15.65	0.84	38.38	2.06	1.19
1938		50	565.5	18.57	15.65	0.84	38.33	2.07	1.19
1906		30	565.6	18.51	15.63	0.85	38.24	2.07	1.18
**SAMPLE DEPTH**									
Deep		19	294.3	18.83	15.69	0.83	38.65	2.05	1.20
Shallow ^b^	102		336.4	18.67	15.67	0.84	38.47	2.06	1.19
**BASELINE ^c^**	8		30.29	18.97	15.71	0.83	38.92	2.05	1.21

^a^ Includes both industrial and commercial land-use. ^b^ Excludes baseline samples. ^c^ Baseline samples were collected only at a shallow depth.

## Data Availability

Data may become available upon request from the authors.

## Supplementary Materials

The following are available online at https://www.mdpi.com/article/10.3390/toxics10060304/s1, Figure S1: Distribution of the ^206^Pb/^204^Pb ratios across different landuse and other categories. The lower and upper boundaries of each box indicate the interquartile range (IQR) of the sample, while the centerline and “X” symbol indicates the sample median and mean, respectively. The lower and upper whiskers indicate the minimum and maximum data points after excluding outliers as defined as Q_1_ or Q_3_±1.5*IQR.
