# Supplementary material for: Use of Radioisotope Ratios of Lead for the Identification of Historical Sources of Soil Lead Contamination in Santa Ana, California
Source: Toxics. 2022 Jun 3;10(6):304. doi: 10.3390/toxics10060304 (PMC9229492; doi:10.3390/toxics10060304)
Supplement: Supplementary file 1 [file toxics-10-00304-s001.zip › toxics-1735230-supplementary.pdf]

toxics

Supplementary Materials: Use of Radioisotope Ratios of Lead for the Identification of Historical Sources of Soil Lead Contamination in Santa Ana, California

Shahir Masri, Alana M. W. LeBrón, Michael D. Logue, Patricia Flores, Abel Ruiz, Abigail Reyes, Juan Manuel Rubio and Jun Wu

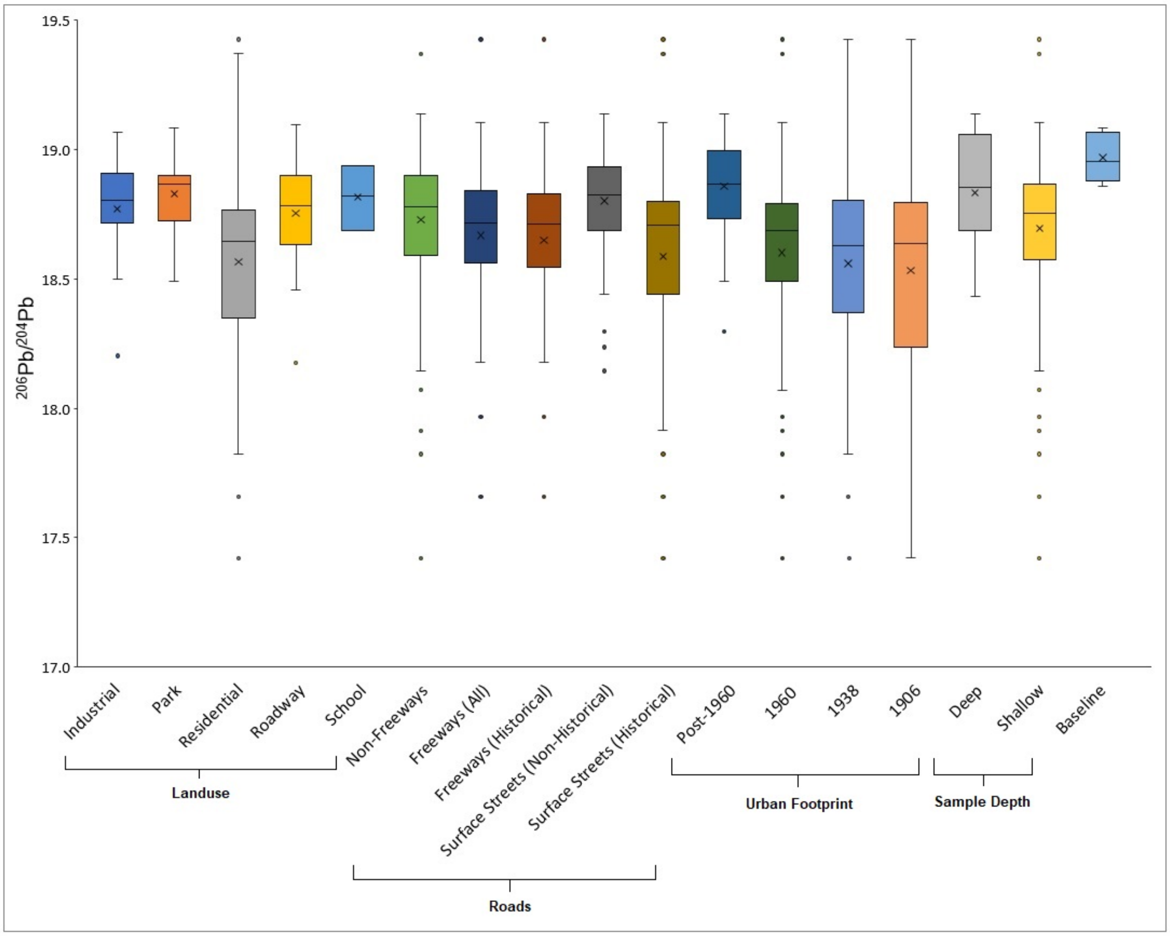

Figure S1. Distribution of the $^{206}Pb/^{204}Pb$ ratios across different landuse and other categories. The lower and upper boundaries of each box indicate the interquartile range (IQR) of the sample, while the centerline and "X" symbol indicates the sample median and mean, respectively. The lower and upper whiskers indicate the minimum and maximum data points after excluding outliers as defined as Q_1 or $Q_3 \pm 1.5$*IQR.
